# Supplementary material for: The maternal drug exposure birth cohort (DEBC) in China
Source: Nat Commun. 2024 Jun 21;15:5312. doi: 10.1038/s41467-024-49623-0 (PMC11192739; doi:10.1038/s41467-024-49623-0)
Supplement: Supplementary file 1 — Supplementary Information [file 41467_2024_49623_MOESM1_ESM.pdf]

The maternal drug exposure birth cohort (DEBC) in China

SUPPLEMENTARY INFORMATION

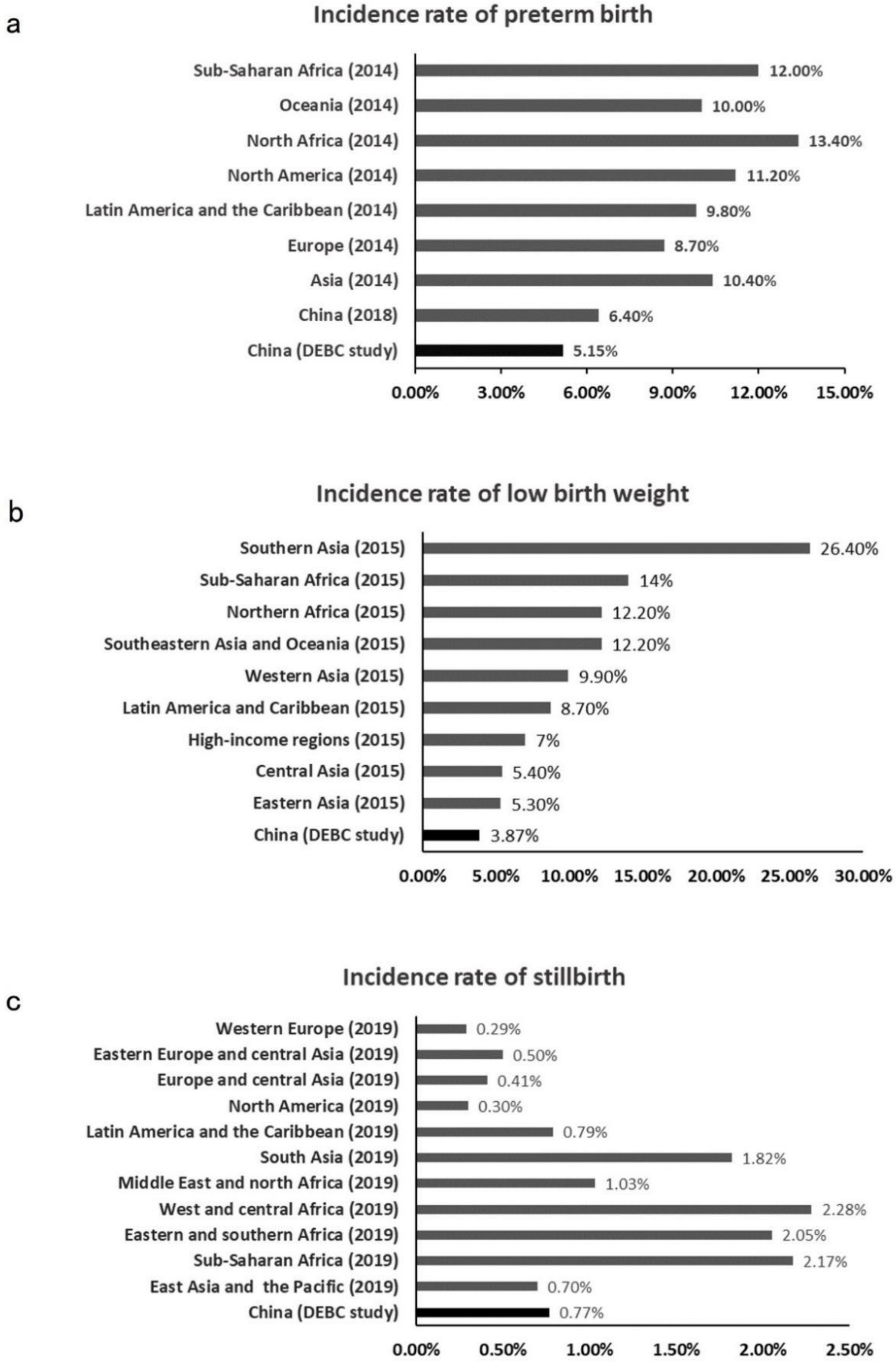

Supplementary Figure 1 Comparison of the incidence rates of adverse birth outcomes observed in the DEBC study with other domestic Chinese investigations and global regions.
a. The incidence rate comparison of preterm birth. b. The incidence rate comparison of low birth weight. c. The incidence rate comparison of stillbirth.

Supplementary Table 1. Comparison of the demographic characteristics between pregnant women without adverse outcomes and with adverse pregnancy outcomes.

**Baseline characteristics**	**Adverse pregnancy outcomes**				***Chi***^{2}
	**NO**		**YES**		
	N	**%**	***N***	**%**	
**Maternal age**					305.814***
<20	945	0.97	171	1.12	
20-24	13447	13.76	1861	12.21	
25-29	39451	40.36	5485	35.99	
30-34	31371	32.09	5105	33.50	
>=35	10605	10.85	2299	15.09	
Missing	1928	1.97	318	2.09	
**Maternal education level**					60.391***
Primary school and below	1137	1.16	216	1.42	
Junior high school	13062	13.36	2343	15.38	
High school	17802	18.21	2752	18.06	
College	59728	61.10	8948	58.72	
Postgraduate and above	5788	5.92	942	6.18	
Missing	230	0.24	38	0.25	
**Fertilization way**					98.625***
Natural conception	92479	94.61	14118	92.64	
Assisted reproduction	4930	5.04	1063	6.98	

*** $P < 0.05$. The P values are two-sided.

Supplementary Table 2. Comparison of the demographic characteristics between initial cohort and DEBC population.

Baseline characteristics	Initial cohort N(%)	DEBC population N(%)	Chi-square value	*P*-value
**Maternal age**			3.1374	0.5351
<20	1575(1.07)	1116(1.01)		
20-24	20341(13.85)	15308(13.82)		
25-29	59625(40.6)	44936(40.58)		
30-34	48366(32.93)	36476(32.94)		
>=35	16963(11.55)	12904(11.65)		
**Maternal education level**			9.5909	0.002
Junior high school and below	16758(14.87)	22980(15.3)		
High school and above	95960(85.13)	127173(84.7)		
**Planned pregnancy**			2.7552	0.0969
Yes	84380(58.89)	66690(59.21)		
No	58910(41.11)	45938(40.79)		
**Folic acid supplementation**			3.7036	0.0543
No	14644(10.32)	11271(10.08)		
Yes	127317(89.68)	100517(89.92)		

The P values are two-sided.

Supplementary Table 3. Specimen collection in DEBC.

Time points	Specimen types (Singleton pregnancy, twins or triplets)			
**First trimester (<14 weeks)**	Peripheral blood	Urine		
**Second trimester (22-26 weeks)**	Peripheral blood	Urine		
**Third trimester (32-36 weeks)**	Peripheral blood	Urine		
**Pregnancy outcomes**				
Miscarriage	Chorionic tissues			
Abortion (< 20 weeks)	Amniotic fluid	Muscular tissues	Pathological tissue	
Abortion (≥20 weeks), Stillbirth	Amniotic fluid	Muscular tissues	Pathological tissue	Placenta
Severe preterm delivery (< 32 weeks)	Placenta	Umbilical cord blood		
Very low birth weight (< 1500g)	Placenta	Umbilical cord blood		
Birth defect	Placenta	Umbilical cord blood		

Supplementary Table 4. The procedures for the specimen collection, processing and storage.

Type	Specimen collection	Specimen aliquot		Collection and pre-treatment	Long-term storage condition
		Type	Number		
**Maternal peripheral blood**	Blood (EDTA) 3-4ml	Plasma	0.5ml×4	centrifuging 10min at 4℃, 3500rpm	-80℃ for years
		Buffy coat	0.5ml×4		
	Blood (Plain) 3-4ml	Serum	0.5ml×4	Standing for 2 hours, then centrifuging 10min at 4℃, 3500rpm	
**Maternal urine**	Urine 20ml	Urine	1ml×4	Not urina sanguinis	-80℃ for years
**Umbilical cord blood**	Blood (EDTA) 3-4ml	Plasma	0.5ml×4	Centrifuging 10min at 4℃, 3500rpm	-80℃ for years
		Buffy coat	0.5ml×4		
	Blood (Plain) 3-4ml	Serum	0.5ml×4	Standing for 2 hours, then centrifuging 10min at 4℃, 3500rpm	
**Amniotic fluid**	Amniotic fluid 8-10ml	Supernatant	0.5ml×4	Centrifuging 10min at 4℃, 1500rpm, and sampling 0.5ml for 4 tubes	-80℃ for years
		Amniocyte	0.5ml×2	Remove excess supernatant, sampling amniocyte 0.5ml for 2 tubes	
**Tissues**	Placenta	Maternal surface	$1.0×1.0×0.5cm^3×4$	On the maternal side, scissoring for 1.0×1.0×0.5cm(length, width, and height) of placenta within 4 quadrants	Liquid nitrogen/-80℃ for years
		Fetal surface	$1.0×1.0×0.5cm^3×4$	On the fetal side, scissoring for 1.0×1.0×0.5cm(length, width, and height) of placenta within 4 quadrants	
	Muscular tissues	Limb muscles	2g×4	Scissoring for 2g muscles of left upper, left lower, right upper, right lower limbs, respectively	
	Birth defect part	Pathological tissue	4-8 pieces	Scissoring for 4-8 pieces in the birth defect part, successively	

Supplementary Table 5. Statistical standard of birth defect

Birth defect	Statistical standard
Anencephaly	Anencephaly with spina bifida, with encephalocele, or with hydrocephalus was counted only as anencephaly. Cephalic malformation and hydranencephaly were excluded.
Spina bifida	Spina bifida with hydrocephalus or spina bifida with encephalocele is only counted as spina bifidus. Spina bifida with anencephaly, spina bifida occulta, and sacrococcygeal teratoma were excluded.
Encephalocele	Encephalocele with spina bifida was excluded.
Congenital hydrocephalus	Hydrocephalus with anencephaly, hydrocephalus with encephalocele, hydrocephalus with encephalocele, and spina bifida should be excluded from congenital hydrocephalus, including hydroanencephaly
Cleft palate	Cleft lip with cleft palate, functional short palate, high and narrow palate and simple cleft palate were excluded.
Cleft lip	Cleft lip with cleft palate and median cleft lip were excluded.
Cleft lip with cleft palate	Simple cleft lip and cleft palate were excluded.
Microtia/Auricularia	Atresia of external auditory canal, dysplasia of auditory canal and low ear with small auricle but normal appearance were excluded.
Other malformations of the external ear	Auricular deformities other than microtia, absence of ears, and/or morphological abnormalities were excluded.
Esophageal atresia or stenosis	Simple esophagotracheal fistula, esophageal ring or web, and tracheoesophageal fistula were included.
Anorectal atresia or stenosis	Ectopic anus, and mild stenosis that did not require surgical correction were excluded.
Hypospadias	Epispadias, penile curvature with a normal urethral opening, phimosis, and pseudohermaphroditism were excluded.
Bladder exstrophy	No special criteria.
Talipes equinovarus	Neonatal talipes varus, multiple joint contracture, spinal deformity, spina bifida, paralytic talipes equinovarus complicated by encephalocele, equinovarus caused by peripheral nerve injury were

	excluded.
Polydactyly	A split nail with a bifurcated deformity of the fingertip was exluded.
Syndactyly	No special criteria.
Limb shortening	Missing fingers (toes) and split hands (split feet) were included; patients with brachydactyly, mild shortening with normal bone structure and shape, syndactyly, and syndesmosis were excluded.
Congenital diaphragmatic hernia	Complete absence of the diaphragm was included; hiatal hernia was excluded.
Omphalocele	Umbilical hernia, gastroschisis and hypoplasia of abdominal wall muscle were excluded.
Gastroschisis	Omphalocele and umbilical hernia were excluded.
Conjoined twins	No special criteria.
Down's syndrome	No special criteria.
Congenital heart disease	Atrial septal defect, ventricular septal defect, transposition of the great vessels, tetralogy of fallot, pulmonary stricture, patent ductus arteriosus, cardiac ectopic, etc, were included.

Supplementary Table 6. ICD10 Codes for Major Birth Defects

Name of deformity	ICD-10 code
**Congenital malformation of nervous system**	
Anencephaly	Q00
Encephalocele	Q01
Microcephaly	Q02
Congenital hydrocephalus	Q03
Spina bifida	Q05
**Congenital malformation of eye, ear, face and neck**	
Congenital cataract	Q12.0
Anophthalmia, microphthalmia, and macrophthalmia	Q11
Congenital absence of auricle	Q16.0
Small ears	Q17.2
Secondary Ear	Q17.0
**Congenital malformation of the circulatory system**	
Ventricular septal defect	Q21.0
Atrial Septal Defect	Q21.1
Tetralogy of Fallot	Q21.3
Hypoplastic left heart syndrome	Q23.4
Patent ductus arteriosus	Q25.0
Transposition great vessels	Q20.3
Pulmonary stenosis	Q25.6
**Cleft lip and palate**	
Cleft palate	Q35
Cleft lip	Q36
Cleft palate with cleft lip	Q37
**Other congenital malformations of the digestive system**	
Esophageal stricture or atresia	Q39.0，Q39.1，Q39.2，

Name of deformity	ICD-10 code
	Q39.3
Anorectal atresia or stenosis	Q42.0，Q42.1，Q42.2，Q42.3
**Congenital malformation of reproductive organ**	
Undescended testicles	Q53
Hypospadias	Q54
Ambiguity and pseudohermaphroditism (hermaphroditism)	Q56
**Other congenital malformations of the urinary system**	
Bladder exstrophy	Q64.1
**Congenital malformation and deformity of musculoskeletal system**	
Talipes equinovarus	Q66.0
Polydactyly	Q69
Syndactyly	Q70
Upper limb deficiency	Q71
Lower limb shortage defect	Q72
Congenital diaphragmatic hernia	Q79.0
Congenital omphalocele	Q79.2
Gastroschisis	Q79.3
**Other congenital malformations**	
Conjoined twins	Q89.4
**Chromosomal abnormalities**	
Down's syndrome	Q90
Trisomy 18 syndrome	Q91
**Hemangioma and lymphangioma**	D18
**Thalassemia**	D56
**Other congenital anomalies**	

Name of deformity	ICD-10 code
Inguinal hernia	K40
Umbilical hernia	K42
Teratoma	D48.9
Congenital phenylketonuria	E70.0
Congenital hypothyroidism	E03

Supplementary References

Annual Report-INTERNATIONAL CLEARINGHOUSE FOR BIRTH DEFECTS SURVEILLANCE AND RESEARCH (ICBDSR)

http://www.icbdsr.org/resources/annual-report/
