# Supplementary material for: The maternal drug exposure birth cohort (DEBC) in China
Source: Nat Commun. 2024 Jun 21;15:5312. doi: 10.1038/s41467-024-49623-0 (PMC11192739; doi:10.1038/s41467-024-49623-0)
Supplement: Supplementary file 3 — Description of Additional Supplementary Files [file 41467_2024_49623_MOESM3_ESM.pdf]

Description of Additional Supplementary Files

Supplementary Software 1: The SAS codes for log-binomial multivariate regression test.
