# Supplementary material for: The maternal drug exposure birth cohort (DEBC) in China
Source: Nat Commun. 2024 Jun 21;15:5312. doi: 10.1038/s41467-024-49623-0 (PMC11192739; doi:10.1038/s41467-024-49623-0)
Supplement: Supplementary file 4 — Supplementary Software 1 [file 41467_2024_49623_MOESM4_ESM.zip › Supplementary software files/legend for Supplemenytary Software 1.docx]

The SAS codes for log-binomial multivariate regression test.
