# Supplementary material for: The maternal drug exposure birth cohort (DEBC) in China
Source: Nat Commun. 2024 Jun 21;15:5312. doi: 10.1038/s41467-024-49623-0 (PMC11192739; doi:10.1038/s41467-024-49623-0)
Supplement: Supplementary file 4 — Supplementary Software 1 [file 41467_2024_49623_MOESM4_ESM.zip › Supplementary software files/variable explanation for code.docx]

The sas file in the supplement files includes the source codes for log-binomial multivariate regression test. The sas file runs well in the SAS 9.4 with Windows 7.

Variables used in models:

| abortion | Miscarriage/Abortion, a category variable. 1 Yes, 0 No. |
| --- | --- |
| stibir | Stillbirth, a category variable. 1 Yes, 0 No. |
| g_preterm | Preterm birth, a category variable. 1 Yes, 0 No. |
| g_lbw | Low birth weight, a category variable. 1 Yes, 0 No. |
| CA1 | Birth defects, a category variable. 1 Yes, 0 No. |
| D084 | Dydrogesterone, a category variable. 1 Yes, 0 No. |
| H056 | Progesterone, a category variable. 1 Yes, 0 No. |
| ZD03 | Fuzheng recipe, a category variable. 1 Yes, 0 No. |
| A056 | Aspirin, a category variable. 1 Yes, 0 No. |
| Z057 | Levothyroxine or thyroxine, a category variable. 1 Yes, 0 No. |
| W089 | Estradiol valerate, a category variable. 1 Yes, 0 No. |
| X035 | Allylestrenol, a category variable. 1 Yes, 0 No. |
| R036 | Chorionic gonadotrophin, a category variable. 1 Yes, 0 No. |
| G047 | Heparin, a category variable. 1 Yes, 0 No. |
| N052 | Menotrophin, a category variable. 1 Yes, 0 No. |
| Q108 | Triptorelin, a category variable. 1 Yes, 0 No. |
| A040 | Amoxicillin, a category variable. 1 Yes, 0 No. |
| G0113 | Ganmaoling granules, a category variable. 1 Yes, 0 No. |
| K059 | Prednisone, a category variable. 1 Yes, 0 No. |
| N051 | Urofollitropin, a category variable. 1 Yes, 0 No. |
| E021 | Dimethylbiguanide, a category variable. 1 Yes, 0 No. |
| X068 | Nifuratel nystatin, a category variable. 1 Yes, 0 No. |
| D0367 | Warming and tonifying kidney-yang recipe, a category variable. 1 Yes, 0 No. |
| K079 | Clotrimazole, a category variable. 1 Yes, 0 No. |
| B0189 | Ban-lan-gen granules, a category variable. 1 Yes, 0 No. |
| J061 | Methylprednisolone, a category variable. 1 Yes, 0 No. |
| T147 | Cefaclor, a category variable. 1 Yes, 0 No. |
| H033 | Cyclosporin, a category variable. 1 Yes, 0 No. |
| dia | a category variable. 1 Yes, 0 No. |
| miscarriage | a category variable. 1 Yes, 0 No. |
| infertility | a category variable. 1 Yes, 0 No. |
| thyroid_dis | a category variable. 1 Yes, 0 No. |
| common_cold | a category variable. 1 Yes, 0 No. |
| vaginitis | a category variable. 1 Yes, 0 No. |
| thrombosis | a category variable. 1 Yes, 0 No. |
| hypertension | a category variable. 1 Yes, 0 No. |
| hepatitis | a category variable. 1 Yes, 0 No. |
| other_inflammatory | a category variable. 1 Yes, 0 No. |
| mage_g | The group of mother’s age(y), a category variable. 1:age<20; 2: 20≤age <25; 3: 25≤age <30; 4: 30≤age <35; 5: 35≤age. |
