# Supplementary material for: The maternal drug exposure birth cohort (DEBC) in China
Source: Nat Commun. 2024 Jun 21;15:5312. doi: 10.1038/s41467-024-49623-0 (PMC11192739; doi:10.1038/s41467-024-49623-0)
Supplement: Supplementary file 5 — Reporting Summary [file 41467_2024_49623_MOESM5_ESM.pdf]

nature portfolio

Corresponding author(s): Ping Yu, Yanping wang, Jun Zhu Last
updated by author(s): May. 16, 2024

Reporting Summary

Nature Portfolio wishes to improve the reproducibility of the work that we publish. This form provides structure for consistency and transparency in reporting. For further information on Nature Portfolio policies, see our Editorial Policies and the Editorial Policy Checkl .

Statistics

For all statistical analyses, confirm that the following items are present in the figure legend, table legend, main text, or Methods section.

n/a	Confirmed	
☐	☒	The exact sample size (*n*) for each experimental group/condition, given as a discrete number and unit of measurement
☐	☒	A statement on whether measurements were taken from distinct samples or whether the same sample was measured repeatedly
☐	☒	The statistical test(s) used AND whether they are one- or two-sided<br>*Only common tests should be described solely by name; describe more complex techniques in the Methods section.*
☐	☒	A description of all covariates tested
☐	☒	A description of any assumptions or corrections, such as tests of normality and adjustment for multiple comparisons
☐	☒	A full description of the statistical parameters including central tendency (e.g. means) or other basic estimates (e.g. regression coefficient) AND variation (e.g. standard deviation) or associated estimates of uncertainty (e.g. confidence intervals)
☐	☒	For null hypothesis testing, the test statistic (e.g. *F*, *t*, *r*) with confidence intervals, effect sizes, degrees of freedom and *P* value noted<br>*Give P values as exact values whenever suitable.*
☒	☐	For Bayesian analysis, information on the choice of priors and Markov chain Monte Carlo settings
☒	☐	For hierarchical and complex designs, identification of the appropriate level for tests and full reporting of outcomes
☐	☒	Estimates of effect sizes (e.g. Cohen's *d*, Pearson's *r*), indicating how they were calculated

Our web collection on statistics for biologists contains articles on many of the points above.

Software and code

Policy information about availability of compute

Data collection	no software was used
Data analysis	All statistical analyses were conducted using STATA® (Version 12.1, StataCorp LLC, College Station, TX, USA) and SAS'" software (Version 9.4, SAS Institute Inc., Cary, NC, USA).

For manuscripts utilizing custom algorithms or software that are central to the research but not yet described in published literature, software must be made available to editors and reviewers. We strongly encourage code deposition in a community repository (e.g. GitHub). See the Nature Portfolio guidelines for submitting co for further information.

Data

Policy information about availability of data

All manuscripts must include a data availability statement. This statement should provide the following information, where applicable:

- Accession codes, unique identifiers, or web links for publicly available datasets
- A description of any restrictions on data availability
- For clinical datasets or third party data, please ensure that the statement adheres to our policy

The raw data is not publicly available due to data privacy laws, according to the China's Ministry of Science and Technology. Individual data should be requested by email to the corresponding author (zhujun028@163.com) and must include the name and full contact information of the person and institution requesting the data, the research objectives, methodology, anticipated outcomes, plans for sharing results, and the purpose of requesting the data. Data requests under agreement will be subjected to appropriate confidentiality obligations and restrictions. Please expect a processing timeframe of approximately 3 months for data requests. Source data are provided with this paper.

Research involving human participants, their data, or biological material

Policy information about studies with human participants or human (. See also policy information about sex, gender (identity/presentation), and sexual orientation and race, ethnicity and racism.

Reporting on sex and gender	As the research subjects in this study were all pregnant women, they were all female, so no sex- and gender-based analyses have been performed
Reporting on race, ethnicity, or other socially relevant groupings	Not relevant to this study
Population characteristics	Population characteristics are maternal age, maternal education level, fertilization way, planned pregnancy, parental smoking, alcohol consumption and folic acid supplementation.
Recruitment	The recruitment was implemented with the cooperation of 49 hospitals at different levels including tertiary or secondary hospitals) from 15 Provinces in China, and Sichuan Province made the major contribution. These medical institutions care for the general population of pregnant women, not a selected population.
Ethics oversight	The DEBC was approved by the Ethics Committee of Sichuan University, China (Protocol ID: K2017045),Written informed

consent" from the patients were collected by the collaborating hospitals of DEBC when the patients were admitted to the hospitals.

Field-specific reporting

Please select the one below that is the best fit for your research. If you are not sure, read the appropriate sections before making your selection.

☒ Life sciences ☐ Behavioural & social sciences ☐ Ecological, evolutionary & environmental sciences

For a reference copy of the document with all sections, see nature.com/documents/

Life sciences study design

All studies must disclose on these points even when the disclosure is negative.

Sample size	According to the cohort study sample size formula, the sample size enrolled in this study was calculated based on the exposure indicator of medication use in early pregnancy (see supplementary method).
Data exclusions	We excluded the pregnant women lost to follow-up, without quality audit or reaching the stage of pregnancy outcomes, or with multiple pregnancies.
Replication	This is an observational study (non-interventional study), therefore the replication of experiments is not involved.
Randomization	This is an observational study (non-interventional study), not relevant to this study
Blinding	This is an observational study (non-interventional study), not relevant to this study

Reporting for specific materials, systems and methods

We require information from authors about some types of materials, experimental systems and methods used in many studies. Here, indicate whether each material, system or method listed is relevant to your study. If you are not sure if a list item applies to your research, read the appropriate section before selecting a response.

Materials & experimental systems

n/a	Involved in the study	
☒	☐	Antibodies
☒	☐	Eukaryotic cell lines
☒	☐	Palaeontology and archaeology
☒	☐	Animals and other organisms
☐	☒	Clinical data
☒	☐	Dual use research of concern
☒	☐	Plants

Methods

n/a	Involved in the study	
☒	☐	ChIP-seq
☒	☐	Flow cytometry
☒	☐	MRI-based neuroimaging

Clinical data

Policy information about clinical studies

All manuscripts should comply with the ICMJE guidelines for publication of clinical research and a completed CONSORT checklist must be included with all submissions.

Clinical trial registration	This is an observational study (non-interventional study), not relevant to this study
Study protocol	The protocol has been briefly described in the method section of the MS, and the detailed protocol is available from the corresponding author on reasonable request.
Data collection	Pregnant women were recruited from forty-nine collaborating medical institutions in fifteen Provinces of China between August, 2018 and December, 2021.
Outcomes	The adverse pregnancy outcomes include miscarriage/abortion, stillbirth, preterm birth, low birth weight, and birth defects

Plants

Seed stocks	*Report on the source of all seed stocks or other plant material used. If applicable, state the seed stock centre and catalogue number. If plant specimens were collected from the field, describe the collection location, date and sampling procedures.*
Novel plant genotypes	*Describe the methods by which all novel plant genotypes were produced. This includes those generated by transgenic approaches, gene editing, chemical/radiation-based mutagenesis and hybridization. For transgenic lines, describe the transformation method, the number of independent lines analyzed and the generation upon which experiments were performed. For gene-edited lines, describe the editor used, the endogenous sequence targeted for editing, the targeting guide RNA sequence (if applicable) and how the editor was applied.*
Authentication	*Describe any authentication procedures for each seed stock used or novel genotype generated. Describe any experiments used to assess the effect of a mutation and, where applicable, how potential secondary effects (e.g. second site T-DNA insertions, mosiacism, off-target gene editing) were examined.*
